# The Effects of Continuous Positive Airway Pressure on Premature Ventricular Contractions and Ventricular Wall Stress in Patients with Heart Failure and Sleep Apnea

**DOI:** 10.1155/2018/2027061

**Published:** 2018-02-07

**Authors:** Sabri Seyis, Adnan Kazım Usalan, Ibrahim Rencuzogullari, Özge Kurmuş, Adil Can Gungen

**Affiliations:** ^1^Department of Cardiology, Live Istinye University Hospital, Istanbul, Turkey; ^2^Department of Chest Disease, Medical Park Hospital, Mersin, Turkey; ^3^Department of Cardiology, Kafkas University Faculty of Medicine, Kars, Turkey; ^4^Department of Cardiology, Ufuk University, Ankara, Turkey; ^5^Department of Chest Disease, Live Istinye University Hospital, Istanbul, Turkey

## Abstract

**Background:**

We aimed to investigate the effects of continuous positive airway pressure (CPAP) treatment on electrocardiography (ECG), premature ventricular contraction load on 24-hour Holter recordings, and implantable cardioverter defibrillator (ICD) shocks in patients with obstructive sleep apnea syndrome (OSAS) and heart failure.

**Methods:**

Patients with heart failure and ICD and patients with newly diagnosed OSAS were divided into two groups according to CPAP treatment. To compare the impact of CPAP on ECG parameters, both baseline and 6-month ECG, 24-hour Holter ECG, ambulatory blood pressure monitoring, echocardiography, polysomnography, and laboratory parameters were collected.

**Results:**

CPAP treatment significantly reduced the frequency of premature ventricular contractions, T-peak to T-end, corrected QT, corrected QT dispersion, and T-peak to T-end/corrected QT ratio in the study group (*p* < 0.001 for all). Although the baseline NT-pro-BNP levels were similar between study and control groups, after six months, the NT-pro-BNP levels of the study group were significantly lower than that of the control group (39.18 ± 7.57 versus 46.11 ± 7.65; *p* < 0.001).

**Conclusions:**

CPAP treatment in patients with heart failure and ICD and in patients with newly diagnosed OSAS may have beneficial effects on premature ventricular contractions and electrocardiographic arrhythmia indices and NT-pro-BNP levels. However, these results are needed to be clarified with further studies.

## 1. Introduction

Obstructive sleep apnea syndrome (OSAS) is characterized by temporary airway collapse during sleep and affects more than 4% of men and 2% of women [1]. Many cardiovascular diseases have been found to be associated with OSAS including arterial hypertension, ischemic heart disease, heart failure, arrhythmias, and stroke. Cardiovascular morbidity and mortality have been shown to be high in patients with OSAS [2, 3]. Sympathetic overactivity, activation of inflammatory cascades, vascular endothelial dysfunction, abnormalities in the coagulation pathway, and metabolic dysregulation are likely to be involved in the pathogenesis of the cardiovascular complications of OSAS.

The relation between the congestive heart failure and OSAS is somewhat complex. OSAS may affect cardiac functions and contribute to the development of heart failure in the long-term. On the other hand, heart failure itself also plays a role in the pathogenesis of OSAS [4–7]. Several studies reported that continuous positive airway pressure (CPAP) treatment in patients with congestive heart failure and OSAS reduced sympathetic activation and improved left ventricular ejection fraction [8–10]. However, the positive effect of CPAP treatment on the cardiovascular system is unclear in terms of the prognosis of patients with congestive heart failure and OSAS [11].

There are some arrhythmic changes on electrocardiography (ECG) in patients with OSAS, and some improvement with CPAP therapy has been reported [12–14]. The association between arrhythmias and OSAS is well known [15, 16]. OSAS associated with complex ventricular ectopia and ventricular tachycardia has been documented in a previous study [17]. CPAP treatment also resulted in a 58% reduction in premature ventricular beats [18]. Those findings may help to explain the pathogenesis of sudden death in OSAS patients [19]. It has also been shown that OSAS patients have a 4-fold higher frequency of atrial fibrillation if apnoea-hypopnoea index (AHI) index is greater than 30 [17].

Heart failure is a life-threatening disease with a growing incidence in developed countries due to better treatment options for heart failure, improvement of survival after myocardial infarction, and increased life expectancy. The introduction of implantable devices such as implantable cardioverter defibrillators (ICD) has improved the overall survival of patients with heart failure [20]. ICD treatment reduces mortality in patients with heart failure. However, certain conditions such as drug-resistant ventricular arrhythmias and atrial fibrillation may increase morbidity and mortality due to inappropriate and frequent shocks.

The aim of the present study is to investigate whether the regular CPAP treatment improves ECG findings and reduces premature ventricular contractions (PVC) and ICD shocks in patients with OSAS, cardiac failure, PVC, and ICD implant.

## 2. Materials and Methods

### 2.1. Study Population

This study is an observational study conducted with patients followed at the Department of Cardiology with diagnosis of chronic heart failure. Our inclusion criteria for the study population were ejection fraction < 35%, stable NYHA class ≥ II, the presence of ICD, sinus rhythm, and >30 PVC/hour on 24-hour Holter monitorization. In addition, patients had to be on stable heart failure medication for at least 4 weeks prior to enrolment and during follow-up. Patients filled Epworth Sleepiness Scale (ESS) form. Clinically suspected patients were referred to Department of Chest Diseases for possible OSAS diagnosis. After polysomnographic study in the sleep laboratory, CPAP treatment was recommended for moderate to severe OSAS patients. Patients only with OSAS were included, and patients with central sleep apnea (CSA) and mixed apnea were not included. Protocol of the study was approved by the local ethics committee, and subjects involved in the study signed informed consent approved by the institution.

Baseline ECG, 24-hour Holter ECG, ambulatory blood pressure monitoring, echocardiography, laboratory investigations, and ICD measurements were recorded. Age, sex, body mass index (BMI), the cause of heart failure, heart rate, arterial blood pressure, left ventricular end diastolic diameter (LVEDD), left atrium (LA) diameter, polysomnography findings, levels of creatinine, and hemoglobin of the participants were also recorded. Class of drugs used by the patient was also recorded. Study group (*n* = 40) consisted of ICD implanted heart failure and OSAS patients who accepted CPAP treatment, and control group (*n* = 40) consisted of ICD implanted heart failure and OSAS patients who did not accept CPAP treatment. Chronic atrial fibrillation, bundle branch block, application of cardiac resynchronization therapy (CRT), the presence of pacemaker rhythm, estimated glomerular filtration rate of less than 60 mL/min/1.73 m^2^ (CKD stage ≥ III), significant lung disease, thyroid dysfunction, and New York Heart Association (NYHA) grade IV heart failure were the exclusion criteria. Six months after the initiation of CPAP therapy, ECG, 24-hour Holter monitorization, ambulatory blood pressure monitoring, and echocardiography were repeated in both groups.

### 2.2. Polysomnography

All referred individuals with heart failure and suspected OSAS were subjected to 1 full-night polysomnography at the sleep laboratory using the Nox A1 PSG System (Nox Medical, Iceland). Physiological variables evaluated during polysomnography included electroencephalogram, electrooculogram, electromyogram (submentonian and tibialis), electrocardiogram, oral/nasal airflow measured by oronasal thermistors, respiratory effort (thorax and abdomen), body position, and blood oxygen saturation (SpO_2_) (mean and minimum SpO_2_). The exam was performed according to specific criteria for the definition of sleep stages [21] by trained technicians who were unaware of the patient characteristics.

Sleep-related respiratory events and arousals were scored according to the American Academy of Sleep Medicine Manual for Scoring Sleep and Associated Events [22]. Central sleep apnea was defined as the absence of thoracic and abdominal movements with absence of airflow. Obstructive sleep apnea was defined as the absence of airflow in the presence of thoracic and abdominal movements. Mixed apnea was defined as apnea without any thoracoabdominal movements in whom thoracoabdominal movements reoccurred prior to reoccurrence of nasal flow. Apnea was defined as complete cessation of the airflow ≥ 10 s. Hypopnea was defined as ≥30% decrease in airflow from baseline for at least 10 seconds with a≥3% oxygen desaturation from pre-event baseline, and/or the event is associated with an arousal. Apnea/hypopnea index was determined via careful calculations. AHI score between 15 and 29 was considered as OSAS with moderate severity. Severe OSAS was defined as AHI ≥ 30.

### 2.3. Treatment

Autotitrating CPAP was used in the sleep laboratory within a period of less than 7 days after the diagnostic study to get a fixed CPAP pressure value. The fixed CPAP pressure was used for the rest of the study. All patients were scheduled for follow-up 2 weeks after randomization and, subsequently, at 4, 8, 12, 18, and 24 weeks. During the follow-up visits, adherence to CPAP and antihypertensive medications was documented. Compliance with CPAP therapy was based on the self-report of patients at the end of the study period. The use ratio of CPAP (%) during the study period, which was the average time with CPAP to total time spent in bed, was 91% (range 72–100%).

### 2.4. Electrocardiography

The 12-lead ECG (CARDIOVIT AT-102 Plus, Schiller, Switzerland) was recorded at a paper speed of 25 mm/s at 10 mm/mV amplitude in the supine position. ECGs were performed while the patient was at rest and at the morning hours (between 8:00 and 10:00 AM). All of the ECGs were scanned with a resolution of 800 dpi and transferred to a personal computer. ECG measurements of QT and Tp-e intervals at V5 were performed by experienced cardiologists who were blinded to the patient data. If V5 was not suitable, V4 or V6 were measured, respectively. A mean value of three readings was calculated for each lead. The QT interval was measured from the beginning of the QRS complex to the end of the T wave. To adjust QT for heart rate, we calculated QTc according to Bazett's formula $\mathrm{QTc}=\mathrm{QT}/\surd \overset{®}{\text{R}}\overset{®}{\text{R}}$, where RR is the RR interval in seconds. The Tp-e interval was defined as the interval from the peak of T wave to the end of T wave. The Tp-e/corrected QT ratio was calculated from these measurements. QTc dispersion is determined as the difference between the maximum and minimum QTc in all considered leads. All ECG measurements were performed by a cardiologist who was blinded to other patient information.

### 2.5. Echocardiographic Evaluation

We used a 2-dimensional, M-mode cardiovascular ultrasound system (Vingmed Vivid 7 system GE, Germany) for echocardiographic evaluation. The examinations were performed in the left lateral decubitus position. Parasternal long- and short-axis views and apical views were used as standard imaging windows. We measured left atrial and left ventricular end systolic diameters from parasternal long-axis view. Ejection fraction was calculated by using modified Simpson method. All echocardiographic examinations were performed by an experienced cardiologist who was blinded to patient data.

### 2.6. Holter Assessment

Holter ECG (medilog®AR4 plus Holter system, Schiller, Switzerland) recordings were analyzed using a MARS 8000 Holter scanner (GE Medical Systems, Milwaukee, Wisconsin). We determined all PVC, atrial fibrillation (AF), and ventricular tachycardia (VT) episodes. The percentage of PVCs was calculated as the total number of ventricular ectopic beats/the total number of beats recorded during 24 hour Holter ECG monitoring.

### 2.7. Ambulatory Blood Pressure Monitoring

Ambulatory blood pressure monitoring was carried for 24 h using an automated sphygmomanometer (BR-102 PLUS PWA, Schiller, Switzerland). The blood pressure was recorded every 20 min during the 24 h period. Mean values were recorded before and after CPAP treatment.

### 2.8. Blood Sampling and Analysis

Fasting blood samples were collected from an antecubital vein in the morning and were centrifuged within one hour. Serum samples were stored at −80°C pending later measurements. N-terminal pro-brain natriuretic peptide (NT-proBNP) was measured by electrochemiluminescent immunoassay (Roche Diagnostics, Basel, Switzerland). Creatinine and hemoglobin levels were measured by standard laboratory methods. Serum NT-proBNP level was measured at baseline and at the end of 6-month follow-up in both groups. The estimated glomerular filtration rate (mL/min per 1.73 m^2^) was calculated to exclude renal dysfunction as a cause for increased NT-proBNP concentrations [23].

### 2.9. Statistical Analysis

All data were entered into a spreadsheet, and statistical analyses were performed by using R 3.3.2v (open source). Data were shown as mean ± standard deviation for continuous variables, median (minimum-maximum) for ordinal ones, and frequency with percent for categorical ones. Comparisons for continuous variables were made by using independent samples *t*-test and for categorical variables Fisher's exact test. Normally distributed variables were compared with paired *t*-test and nonnormally distributed variables were compared with Mann–Whitney *U* test for intragroup and intergroup differences. Two-Way Repeated Measures of ANOVA was also used for intragroup comparisons. A *p* value less than 0.05 was considered statistically significant.

## 3. Results

The mean age of the study group was 58.4 ± 8.14 years and the control group was 59.18 ± 7.97 years. The female/male ratio in the control group was 8/32 and in the control group was 9/31. There was no significant difference between the groups in terms of age and sex (*p*=0.67 and *p*=0.79). Baseline characteristics and other demographics of the study and control groups are summarized in Table 1.

Other baseline characteristics including BMI, ejection fraction, AHI, oxygen saturation, heart rate, and blood pressure were also similar between both groups (*p* > 0.05 for all). Drugs used by the patients in both groups are summarized in Table 2. Drug usage characteristics of the study and control groups were also similar. No medicational change was done during follow-up especially in terms of antiarrhythmic drugs.

ESS values were similar between both groups (10.51 ± 4.82 versus 10.73 ± 5.23, *p*=0.74).

At the end of 6 months of CPAP treatment, frequency of PVC, Tp-e, QTc, QTc dispersion, and Tp-e/QTc ratio decreased significantly in the study group (*p* < 0.001 for all). These parameters did not change significantly in the control group after 6 months (*p* > 0.05 for all).  Table 3 summarizes the chances of frequency of PVC, Tp-e, QTc, QTc dispersion, and Tp-e/QTc ratio during the follow-up period in both groups.

CPAP treatment significantly reduced the heart rate and systolic and diastolic blood pressure and increased the ejection fraction in the study group (*p* < 0.05 for all). BMI and LVEDD did not change significantly after 6 months of CPAP treatment.  Table 4 summarizes the BMI, ejection fraction, heart rate, blood pressure, and LVEDD measures of the groups in detail.

Appropriate ICD shocks during follow-up in study and control groups due to VT/VF were seen in 5 patients and 9 patients, respectively (%12.5 versus %22.5, *p*=0.24). Inappropriate ICD shocks were much more in control group (%2.5 versus %7.5, *p*=0.62) but did not reach statistical significance. Also atrial fibrillation rate detected by ICD devices was higher in control group but again did not have statistical significance.

**Figure 1:**
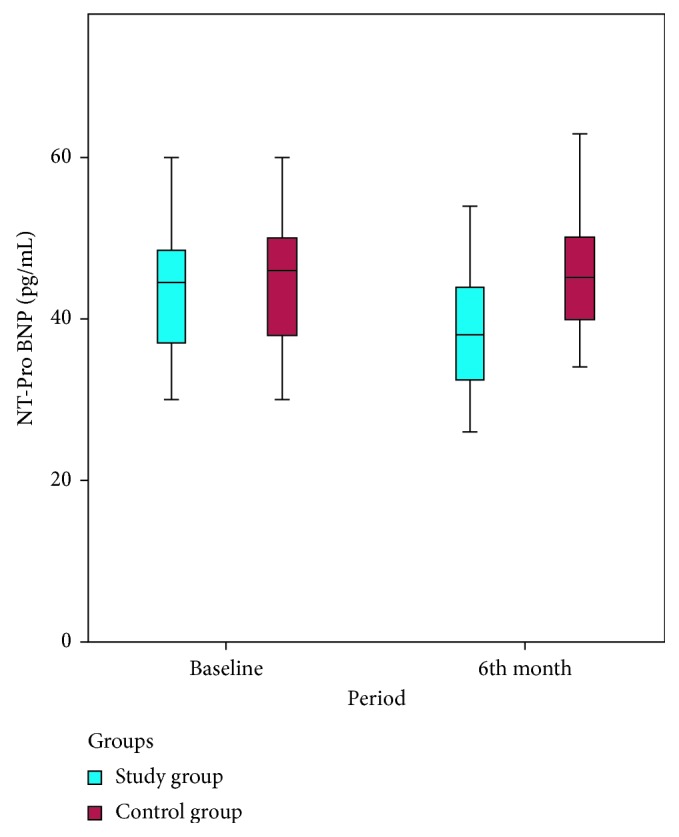
The baseline and 6-month NT-pro BNP levels in both groups.

Baseline NT-Pro BNP levels were 43.88 ± 7.79 pg/mL in the study group and 44.65 ± 7.79 pg/mL in the control group. The NT-Pro BNP levels at 6th month were 39.18 ± 7.57 pg/mL in the study group and 46.11 ± 7.65 pg/mL in the control group. There was a statistically significant difference between baseline and 6th-month measurements in the study group in terms of NT-Pro BNP (*p* < 0.001). There was also a statistically significant difference between the study and control groups at 6th month (*p* < 0.001), but not baseline (*p*=0.112) NT-Pro BNP measurements.  Figure 1 shows the baseline and 6th-month NT-pro-BNP levels in the groups.

## 4. Discussion

Individuals with OSAS have frequent cessation or reduction of airflow during sleep that results in hypoxemia and arousals from sleep. Continuous positive airway pressure (CPAP) is the standard first-line treatment for OSAS [24]. One metaanalysis from consistent evidence from good- and fair-quality randomized controlled trials reported that CPAP effectively reduces AHI and excessive sleepiness in patients with OSAS [25]. Several studies showed the beneficial effects of CPAP on cardiac functions in OSAS patients [26, 27]. The possible mechanism of action of CPAP may include improved myocardial oxygen delivery, decreased sympathetic activity, left ventricular transmural pressure, and afterload [28]. In the present study, we have found beneficial effects of CPAP treatment in patients with OSAS and heart failure in terms of reduction in premature ventricular contractions and ventricular wall stress.

The issue of whether OSAS is an independent risk factor for patients with heart failure is controversial. The results of published studies on this subject vary significantly [29, 30]. Lanfranchi et al. [29] reported approximately 4-fold increased mortality rates in patients with OSAS and heart failure compared to heart failure patients without OSAS. Fries et al. [30] investigated the prognostic value of OSAS in congestive heart failure patients with ICD implants and found that mortality rates were 44% in central sleep apnea and 13% in the absence of apnea at the 2 years of follow-up (*p* < 0.05), but this study found no association between sleep apnea and appropriate ICD therapy for VT or VF. Adlakha and Shepard [31] speculated that hypoxia in the obstructive sleep apnea leads to cardiac arrhythmias. Sudden deaths, particularly at night, in OSAS may be due to these arrhythmias [19, 32]. Clay et al. [33] reported that OSAS patients with ICD had higher rates of ventricular ectopia and nonsustained ventricular tachycardia. Javaheri [34] found an increased frequency of VT and VES in patients with congestive heart failure and OSAS. Bitter et al. [35] speculated that OSAS is an independent risk factor for malignant ventricular arrhythmias that require ICD treatment. We found reduced premature ventricular contraction rates after 6 months of CPAP treatment in patients with OSAS and heart failure compared to untreated controls. We did not make major changes in the heart failure treatment of patients. So, favorable results would be attributable to CPAP treatment.

Ventricular repolarization and arrhythmogenesis can be assessed by several electrocardiographic parameters including QTc interval, QT dispersion, and T wave measurements [36]. Tp-e is used as an indicator of transmural dispersion of ventricular repolarization [37]. Prolonged Tp-e interval may predict ventricular arrhythmias and mortality [38, 39]. Thus, Tp-e/QTc ratio has been proposed to be a more specific index of ventricular arrhythmogenesis [40, 41]. Kilicaslan et al. [36] reported prolonged Tp-e interval and increased Tp-e/QTc ratio in patients with moderate and severe OSAS as compared to healthy controls. Their finding of Tp-e and QTc interval in OSAS patients were comparable with the present study results. We found that, CPAP treatment significantly reduced the frequency of PVC, Tp-e interval, QTc interval, QTc dispersion, and Tp-e/QTc ratio in the study group, whereas the control group did not show any significant difference for the mentioned parameters.

Myocytes of cardiac ventricle constitute the major source of BNP-related neurohormones. B-type natriuretic peptide (BNP) is a 32 amino acid peptide primarily synthesized, stored, and secreted from left ventricle. NT proBNP is derived from the BNP and secreted when the ventricular wall stress increased [42, 43]. It has been used as an indicator of heart failure and symptomatic idiopathic PVC [42, 44, 45]. NT proBNP suggested as a sensitive indicator of PVC-induced increased ventricular wall stress [44, 45]. Skranes et al. [46] reported that the higher levels of NT-proBNP levels are independently associated with the incidence of frequent ventricular ectopy and complex ventricular ectopy in a moderately large, community-based population. Another study [47] found that fatigue was associated with higher baseline NT-proBNP in patients with frequent PVCs and preserved LV function. Tasci et al. [48] reported that nasal CPAP treatment significantly reduced NT-proBNP in patients with normotensive and hypertensive OSAS. Similarly, Strehmel et al. reported reduced NT-proBNP levels after CPAP treatment in patients with OSAS and coronary artery disease [49]. In the present study, we have found that 6 months CPAP treatment for OSAS significantly reduced the NT-proBNP levels which means reduced ventricular wall stress.

The present study has some limitations. We did not evaluate the AHI index using polysomnography at the 6-month follow-up. Therefore, an association between AHI and cardiac functions remained unclarified. The randomization in this study depended on the patient preferences (whether or not CPAP treatment is desired). However, real randomization in this context carries ethical considerations. The relatively small number of patients in our study is another limitation. A larger patient population with a longer follow-up would provide more precise results.

## 5. Conclusions

We have found that the frequency of PVC and NT-proBNP levels can be reduced by CPAP treatment in patients with heart failure and OSAS. CPAP therapy has been shown to significantly improve cardiac functions. Further studies are needed to clarify the possible relationships between CPAP and cardiac functions in detail.

## Figures and Tables

**Table 1:** Baseline characteristics and other demographics of the study and control groups.

	Groups		
	Study group	Control group	*p* value
	(*n* = 40) (%)	(*n* = 40) (%)	
Age (years)	58.4 ± 8.14	59.18 ± 7.97	0.67
Gender			
Female	8 (20)	9 (22.5)	0.79
Male	32 (80)	31 (77.5)	
Body mass index (kg/m)	28.63 ± 3.16	27.88 ± 2.63	0.25
Cause of congestive heart failure			
Ischemic	32 (80)	28 (70)	0.30
Nonischemic	8 (20)	12 (30)	
Primary or secondary ICD			
ICD for primary protection	25 (62.5)	31 (77.5)	0.14
ICD for secondary protection	15 (37.5)	9 (22.5)	
Polysomnography characteristics			
Apnea/hypopnea index	35.85 ± 8.61	32.45 ± 8.88	0.09
Total sleep time (minutes)	341.75 ± 45.45	337.65 ± 45	0.69
Arousals/h sleep time	29.25 ± 8.18	28.73 ± 8.95	0.79
Mean O_2_ saturation (%)	93.08 ± 1.27	93.35 ± 1.35	0.35
Minimum O_2_ saturation (%)	78.98 ± 2.9	79.95 ± 2.91	0.14
Epworth sleepiness scale	10.51 ± 4.82	10.73 ± 5.23	0.74
Heart rate (beats per minute)	68.3 ± 6.46	68.7 ± 6.31	0.78
Blood pressure (mmHg)			
Systolic	131.13 ± 7.02	128.25 ± 7.39	0.08
Diastolic	76.5 ± 8.02	76 ± 7.18	0.77
Echocardiography characteristics			
Ejection fraction (%)	30.2 ± 4.36	30.78 ± 4.7	0.57
Left ventricular end diastolic diameter (mm)	61.53 ± 4.31	61.68 ± 4.7	0.88
Left atrium diameter (mm)	39.25 ± 3.18	39.5 ± 3.35	0.73
Creatinine (mg/dL)	1.21 ± 0.28	1.17 ± 0.25	0.42
Hemoglobin (g/dL)	12.22 ± 2.03	12.45 ± 1.06	0.54
Atrial fibrillation			
No	39 (97.5)	37 (92.5)	0.62
Yes	1 (2.5)	3 (7.5)	
ICD shock appropriate			
No	35 (87.5)	31 (77.5)	0.24
Yes	5 (12.5)	9 (22.5)	
ICD shock inappropriate			
No	39 (97.5)	37 (92.5)	0.62
Yes	1 (2.5)	3 (7.5)	

**Table 2:** Drug usage characteristics of the groups.

	Groups		*p* value
	Study group (*n* = 40)	Control group (*n* = 40)	
*β* blocker			
Not using	3 (7.5)	3 (7.5)	1.0
Using	37 (92.5)	37 (92.5)	
Renin-angiotensin system blocker			
Not using	2 (5)	2 (5)	1.0
Using	38 (95)	38 (95)	
Aldosterone antagonist			
Not using	9 (22.5)	9 (22.5)	1.0
Using	31 (77.5)	31 (77.5)	
Class III antiarrhythmic agents			
Not using	25 (62.5)	30 (75)	0.23
Using	15 (37.5)	10 (25)	
Diuretics			
Not using	5 (12.5)	3 (7.5)	0.71
Using	35 (87.5)	37 (92.5)	

**Table 3:** The chances of frequency of PVC, Tp-e, QTc, QTc dispersion, and Tp-e/QTc ratio during the follow-up period in both groups.

	Study group			Control group			Study group versus control group	
							Baseline	6th month
	Baseline	6th month	*p* value^∗^	Baseline	6th month	*p* value^∗^	*p* value	*p* value
Frequency of PVC	1290 (865–1665)	945 (685–1330)	<0.001	1360 (960–1765)	1325 (990–1740)	0.57	0.82	0.004^∗∗^
Tp-e interval (ms)	79.7 ± 7.02	71.85 ± 6.07	<0.001	79.18 ± 8.01	79.13 ± 7.97	0.89	0.76	<0.001
QTc interval (ms)	410.05 ± 11.2	397.2 ± 11.64	<0.001	410.43 ± 12.11	414.25 ± 11.56	0.004	0.89	<0.001
QTc dispersion	53.28 ± 8.51	37.95 ± 8.31	<0.001	55.48 ± 9.97	54.83 ± 9.58	0.16	0.29	<0.001
Tp-e/QTc ratio	194.18 ± 14.99	180.48 ± 13.89	<0.001	192.15 ± 15.03	190.48 ± 16.81	0.13	0.55	<0.001

^∗^Two-way repeated measures ANOVA was used; ^∗∗^frequency of premature ventricular contractions were compared between the groups using Mann–Whitney *U* test; PVC: premature ventricular contractions; Tp-e: T-peak to T-end interval; QTc: corrected QT interval.

**Table 4:** The comparisons of certain parameters of the groups during the follow-up period.

	Study group			Control group		
	Baseline	6th month	*p* value	Baseline	6th month	*p* value
Body mass index (kg/m^2^)	28.63 ± 3.16	28.65 ± 2.92	0.812	27.88 ± 2.63	27.83 ± 2.54	0.534
Ejection fraction (%)	30.2 ± 4.36	30.98 ± 4.19	**<0.001**	30.78 ± 4.7	30.75 ± 4.49	0.860
Heart rate (beats per minute)	68.3 ± 6.46	67.38 ± 6.59	**0.002**	68.7 ± 6.31	68.48 ± 6.26	0.060
Systolic blood pressure (mmHg)	131.13 ± 7.02	127.5 ± 5.66	**<0.001**	128.25 ± 7.39	127.75 ± 7.16	0.160
Diastolic blood pressure	76.5 ± 8.02	74.13 ± 6.59	**<0.001**	76 ± 7.18	76 ± 7.18	0.999
Left ventricular end diastolic diameter (mm)	61.53 ± 4.31	61.68 ± 4.32	0.262	61.68 ± 4.7	62.03 ± 4.65	**0.001**
